# Improved survival for sequentially as opposed to concurrently delivered neoadjuvant chemotherapy in non-metastatic breast cancer

**DOI:** 10.1007/s10549-017-4364-8

**Published:** 2017-07-03

**Authors:** B. E. P. J. Vriens, I. J. H. Vriens, M. J. B. Aarts, S. M. van Gastel, F. W. P. J. van den Berkmortel, T. J. Smilde, L. J. C. van Warmerdam, D. J. van Spronsen, P. G. M. Peer, M. de Boer, V. C. G. Tjan-Heijnen, on behalf of the Breast Cancer Trialists’ Group of the Netherlands (BOOG)

**Affiliations:** 1 Division of Medical Oncology, Department of Medical Oncology, GROW-School for Oncology and Developmental Biology, Maastricht University Medical Centre, P.O. Box 5800, 6202 AZ Maastricht, The Netherlands; 2 Comprehensive Cancer Centre the Netherlands, Nijmegen, The Netherlands; 3 Department of Internal Medicine, Zuyderland Medical Centre, Heerlen, The Netherlands; 4 Department of Internal Medicine, Jeroen Bosch Hospital, ’s Hertogenbosch, The Netherlands; 5 Department of Internal Medicine, Catharina-Hospital, Eindhoven, The Netherlands; 6 Department of Internal Medicine, Canisius-Wilhelmina Hospital, Nijmegen, The Netherlands; 7 Biostatistics, Radboud Institute for Health Sciences, Nijmegen, The Netherlands

**Keywords:** Breast cancer, Neoadjuvant chemotherapy, Disease-free survival, Overall survival

## Abstract

**Purpose:**

The INTENS study was designed to determine whether delivering neoadjuvant chemotherapy at a higher dose in a shorter period of time improves outcome of breast cancer patients.

**Methods:**

Women with newly diagnosed breast cancer were randomly assigned to neoadjuvant chemotherapy consisting of four cycles of doxorubicin and cyclophosphamide followed by four cycles of docetaxel (AC 60/600–T 100 mg/m^2^) or six cycles of TAC as triplet chemotherapy (75/50/500 mg/m^2^) every 3 weeks. The primary outcome was the pathologic complete response (pCR), with disease-free and overall survival as secondary endpoints.

**Results:**

In total, 201 patients were included. The pCR rates were 28% for patients treated with AC-T and 19% for patients treated with TAC, with an odds ratio of 1.60 (95% CI 0.90–3.21). With a median follow-up of 6 years (range 0.04–8.41 years), the five-year disease-free survival was 81% for patients treated with sequentially AC-T and 71% for patients treated with concurrent triplet TAC chemotherapy with a stratified hazard ratio (HR) of 0.50 (95% CI 0.29–0.86). Five-year overall survival was 84% versus 76%, respectively, with a stratified HR of 0.55 (95% CI 0.29–1.03).

**Conclusions:**

No differences were observed between the two treatment arms with respect to pCR rate, but the sequentially delivered chemotherapy outperformed the triplet combination chemotherapy in terms of survival, despite a lower cumulative dose per agent. GOV nr NCT00314977.

## Introduction

It is accepted worldwide that taxanes should somehow be incorporated in the (neo)adjuvant treatment of breast cancer patients at increased risk of relapse. The most optimal strategy is however, still under investigation. Previously, we reported the breast pathological complete response (pCR) results from our Dutch phase III breast cancer study (the INTENS trial) comparing sequential versus concurrent use of taxanes in addition to doxorubicin and cyclophosphamide [1].

To support accelerated approval, pCR in breast cancer is formally approved by the Food and Drug Administration as a surrogate endpoint of neoadjuvant chemotherapy trials for efficacy and to prognosticate long-term outcomes, especially in high risk patients (triple negative, HER2 (human epidermal growth factor receptor) positive) [2]. Apparently, there is an interplay between treatment efficacy and tumour biology resulting in differential outcome patterns, as was shown in more recent neoadjuvant trials [3–5]. The prognostic value of pCR and long-term outcome seemed to be strongest in patients with more aggressive subtypes [3–5].

In the present analysis of the INTENS trial, we report the disease-free and overall survival rates by treatment arm, presence or absence of pCR and the effect of treatment per breast cancer subtype.

## Patients and methods

The study design, patient characteristics and pCR results have been reported before [1].

### Study design

In short, the INTENS trial is a Dutch phase III study in which patients were randomly allocated to neoadjuvant chemotherapy in a sequential schedule consisting of four cycles of doxorubicin and cyclophosphamide followed by four 3-weekly cycles of docetaxel (AC-T; 60, 600 and 100 mg/m^2^, respectively) or six 3-weekly cycles of a concurrent schedule consisting of the same drugs at a different dose per cycle (TAC; 75, 50 and 500 mg/m^2^, respectively) and different cumulative dose and dose-intensity per drug for the entire schedule. At the time, HER2-targeted therapy was used as adjuvant treatment, also in patients with HER2-positive disease who were treated with neoadjuvant chemotherapy. The study was conducted in accordance with the Declaration of Helsinki and the principles of Good Clinical Practice.

### Patients

Patients with non-metastatic breast cancer and a Karnofsky Performance Score of at least 70 with a clinical tumour size of at least 3 cm and/or positive regional lymph nodes were eligible. A total of 201 assessable patients were included from February 2006 through April 2009 from 21 hospitals in the Netherlands.

### Study endpoints and statistical analyses

The primary outcome measure of the Dutch INTENS study was the pCR rate after neoadjuvant chemotherapy, defined as postoperative absence of invasive tumour in the breast. To achieve 80% power at a 5% level of significance for the detection of a difference in proportion of pCR of 16% versus 34%, a total of 180 eligible patients were required. These percentages were based on results from the Aberdeen trial [6]. Taking a 10% drop out into account, it was decided to enrol a total of 200 patients. The results on the primary endpoint have been reported before [1].

The primary objective of the current study was to determine the outcome in terms of disease-free survival and overall survival according to treatment arm. Disease-free survival was defined as time from date of randomization until the date of occurrence of local or regional recurrence, contralateral or second primary ipsilateral breast cancers (including DCIS) or death of any cause. Overall survival was defined as time from the date of randomization until date of death of any cause. All patients still alive were censored at the date of last follow-up of each individual patient.

All analyses were done on the intent-to-treat population. Survival curves were obtained using the Kaplan–Meier method and tested for differences between two groups with the log-rank test.

The impact of treatment was expressed in a hazard rate ratio obtained in a Cox-model stratified for clinical tumour stage cT1-2 and cT3-4, clinically nodal status cN-negative (cN0) and cN-positive (cN+), receptor status (ER, oestrogen; PR, progesterone) and HER2-status. The impact of treatment was also assessed in specific patients groups (age ≤50 years and age >50 years).

All reported P-values are two-sided and *P* value <0.05 was considered statistically significant. The 95% confidence intervals (95% CI) were given whenever appropriate.

## Results

### Patient characteristics and treatment

Characteristics in terms of demographics and tumour were well balanced across the groups (Appendix Table 1). At enrolment, median age was 49 years (range, 24–70 years). Many patients had rather extensive locoregional disease, with nearly 50% of them having cT3-4 tumours and 75% having clinical involvement of axillary lymph nodes, 66% of patients had ER and/or PR-positive disease, 20% HER2-positive disease and 25% triple-negative disease [1].

**Table 1:** Baseline patient and tumour characteristics

	AC-T (*N* = 100) %	TAC (*N* = 101) %
Age, years		
Median	49	49
Range	27–70	24–68
Initial tumour status		
cT1-cT2	51	50
cT3-4	49	50
Initial nodal status		
cN0	24	26
cN+	76	74
Receptor status		
HR+/HER2−	51	58
HR+/HER2+	15	8
HR−/HER2+	11	8
HR−/HER2−	23	27

*A* doxorubicin, *C* cyclophosphamide, *T* docetaxel, *HR* hormone receptor, *HER2* human epidermal growth factor receptor 2

### Pathologic complete response rate

Based on the local pathology reports, the pCR rates were 28 and 19%, respectively, with an odds ratio of 1.60 (95%CI 0.90–3.21) [1].

### Disease-free and overall survival per treatment arm and disease-free survival per stratum

**Fig. 1:**
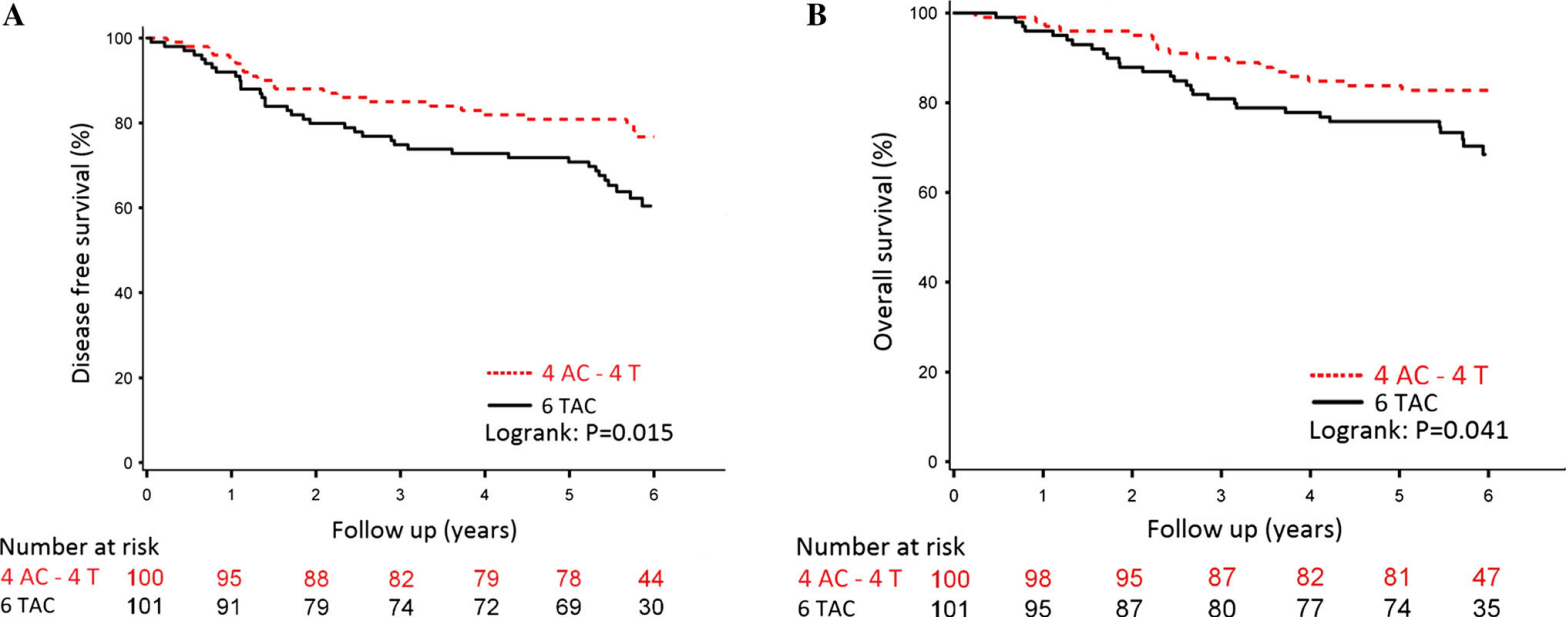
Disease-free (**a**) and Overall Survival (**b**) per neoadjuvant treatment arm: four cycles of doxorubicin and cyclophosphamide followed by four cycles of docetaxel (AC-T) or six cycles of concurrent triplet chemotherapy (TAC)

After a median follow-up of 6 years (range 0.04–8.41 years), 5-year disease-free survival was 81% for patients treated with sequential AC-T chemotherapy and 71% for patients treated with concurrent triplet TAC chemotherapy (log-rank *P* = 0.015), resulting in a stratified HR of 0.50 (95% CI 0.29–0.86) in favour of the sequential treatment arm (Fig. 1a). Five-year overall survival was 84% for the patients treated with AC-T chemotherapy versus 76% for those treated with TAC chemotherapy, resulting in a stratified HR of 0.55 (95% CI 0.29–1.03) (Fig. 1b).

**Fig. 2:**
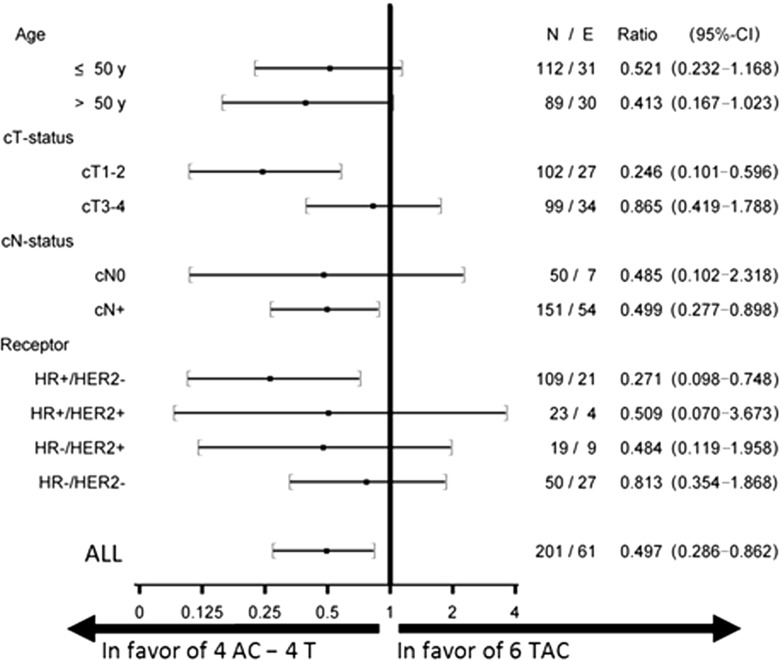
Forest plots comparing groups with AC-T and those treated with TAC neoadjuvant chemotherapy within various subsets for disease-free survival. The hazard ratios (HR) and their 95% CIs are obtained from the corresponding Cox proportional hazards models. HR < 1 implies benefit from AC-T when compared to TAC. *HR* hormone receptor, *HER2* human epidermal growth factor receptor 2

Sequential treatment provided the largest disease-free survival benefit in patients with cT1-2 tumours (HR 0.25; 95% CI 0.10–0.60) and hormone receptor-positive/HER2-negative disease (HR 0.27; 95% CI 0.10–0.75) (Fig. 2).

### Outcome related to pCR in the breast

**Fig. 3:**
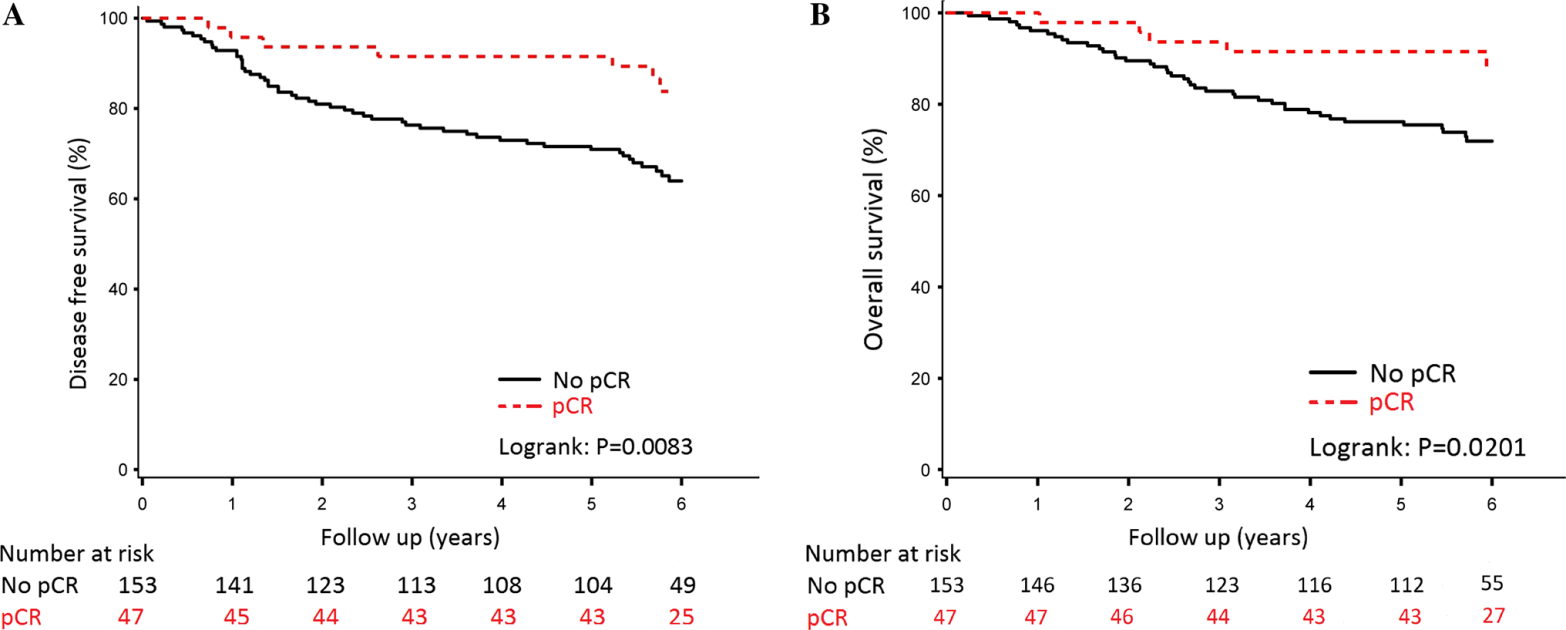
Disease-free Survival (**a**) and Overall survival (**b**) stratified by pCR for the overall population, irrespective of chemotherapy schedule

**Fig. 4:**
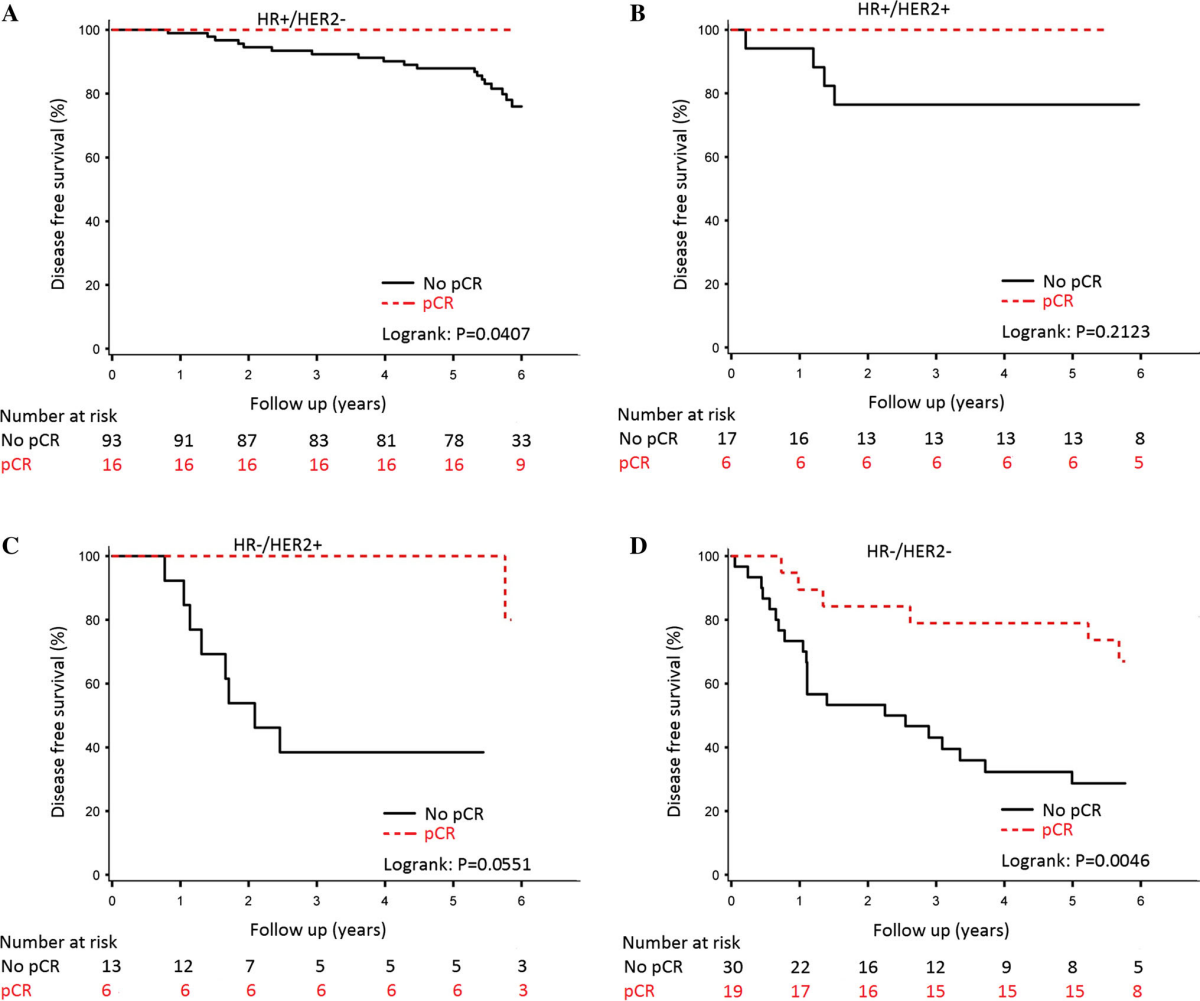
Disease-free survival stratified by pCR for the overall population per tumour subtype: hormone receptor (HR)/human epidermal growth factor receptor 2 (HER2), positive (+) and negative (−) subsets

Five-year disease-free survival was 91% for those having a pCR in the breast and 71% for those without pCR (log-rank *P* value = 0.008) (Fig. 3). In Fig. 4, the results are shown by tumour subtype: hormone receptor-positive/HER2-negative (Fig. 4a: log-rank *P* value = 0.041), hormone receptor-positive/HER2-positive (Fig. 4b: log-rank *P* value = 0.212), hormone receptor-negative/HER2-positive (Fig. 4c: log-rank *P* value = 0.055) and hormone receptor-negative/HER2-negative (Fig. 4d: log-rank *P* value = 0.0046).

Comparable results were obtained for overall survival (not further shown).

## Discussion

In this Dutch phase III neoadjuvant chemotherapy study, breast cancer patients were treated by either four cycles of doxorubicin and cyclophosphamide followed by four cycles of docetaxel (AC-T) or six cycles of concurrent triplet chemotherapy (TAC) [1]. Previously, we reported the results on pCR based on the local pathology reports [1]. The pCR rates were 28% for patients treated with AC-T and 19% for patients treated with TAC, with an odds ratio of 1.60 (95%CI 0.90–3.21).

Now, we report the results after a median follow-up of six years, showing a superior disease-free and overall survival with sequentially delivered AC-T chemotherapy. Notably, all patient subgroups benefitted from the sequentially delivered eight cycles of treatment as compared to those treated with the triplet schedule, but the size of the benefit differed. Specifically patients with more favourable tumour characteristics benefitted the most, with a HR of 0.25–0.27 for disease-free survival. Moreover, we noticed that patients with a pCR and hormone receptor-positive disease had an excellent 5-year disease-free survival.

Both the adjuvant breast cancer BIG 2-98 and the NSABP-B30 trials compared sequential versus concurrent use of taxanes resulting in a better disease-free survival for the sequential arm [7, 8]. The BIG 2-98 trial compared amongst others six cycles of sequentially delivered A-T with four cycles of concurrently delivered AT chemotherapy, both after treatment with CMF (cyclophosphamide, methotrexate, 5-fluorouracil) chemotherapy, resulted in a HR of 0.83 (95% CI 0.69–1.00) for disease-free survival in favour of the sequential treatment arm [7]. The NSABP-B30 trial compared AC-T for eight cycles to TAC for four cycles again showed an improved disease-free survival for the sequential arm at a hazard ratio of 0.83 (95% CI 0.73–0.95) [8]. In contrast, in the large adjuvant BCIRG-005 trial, eight cycles of AC-T did not improve disease-free survival when compared to six cycles of TAC (HR 1.00; 95% CI 0.86–1.16) [9]. In a recent meta-analysis, it was shown that patients with hormone receptor-negative breast cancer may benefit from dose-dense chemotherapy, whereas those with hormone receptor-positive breast cancer did not [10]. Apparently, number of chemotherapy cycles, dose per cycle and frequency of chemotherapy delivery all matter. In our study, each drug was given at a higher dose per cycle (dose-intensity), but at a lower cumulative dose at a 3-weekly interval. Taking all the results into account, we can at least conclude that sequentially delivered chemotherapy was always superior or comparable, but never inferior, to concurrently delivered triplet chemotherapy. Therefore, sequentially delivered chemotherapy may thus be the preferred treatment strategy for non-metastatic breast cancer.

The patients in the NSABP-B18 study received a combination of doxorubicin and cyclophosphamide (AC) chemotherapy every 3 weeks [11]. The investigators of this trial were the first to show that patients with a pCR in the breast had an improved 5-year disease-free survival, suggesting its value as prognosticator [11, 12]. Unexpectedly, an improvement in pCR rate by the addition of neoadjuvant docetaxel to AC chemotherapy did not result in a further improved overall survival in the NSABP-B27 trial [13]. In our study, addressing all biomarker subtypes, there is a non-significant pCR improvement for AC-T versus TAC. More than half of the patients in our study had ER-positive and HER2-negative disease, this is the group excluded from the accelerated FDA approval. We conclude that in this population with the majority of patients with ER-positive and HER2-negative disease, a longer follow-up period is necessarily to take conclusions about survival because pCR is a poor predictor of clinical benefit in this population and drug-efficacy may be overall underestimated. This is in line with other studies that despite the lower pCR rates in this population, patients with hormone receptor-positive tumours nonetheless have a more favourable long-term prognosis [5].

In our study, patients with pCR in the breast had a significantly better survival than those without pCR (Fig. 3). Although numbers per subgroup were too low to draw firm conclusions, it was noted that patients who did obtain a pCR in the breast with ER and/or HER2-positive disease had an excellent outcome (Fig. 4). Of note, patients with HER2-positive disease (20% of all) received trastuzumab only as adjuvant treatment, which may be considered a limitation of the study and may also explain the lack of significance between pCR and disease-free and overall survival for the HER2-positive subgroups. Patients with triple-negative breast cancer and pCR in the breast had a significantly better outcome than those without pCR, 80% 5-year disease-free survival for patients who had a pCR versus 30% for those without pCR. These results are in line with the meta-analysis of Cortazar, although they reported a modest higher 5-year event-free survival of approximately 50% in patients with triple-negative breast cancer with failure to achieve pCR [5]. The inferior outcome in our study may be explained by inclusion of more patients with aggressive characteristics as cT3-4 tumours and clinically node-positive tumours.

The BIG 2-98 study showed that there is an association between presence of tumour infiltrating lymphocytes and prognosis [14]. Moreover, patients with HER2-positive disease with increased stromal lymphocytic infiltration had a larger benefit of anthracycline-based therapy compared to those receiving combination anthracycline–docetaxel therapy. These results suggest that specific chemotherapy schedules in specific breast tumours may trigger the immune system which contributes to treatment efficacy. Indeed, induced cancer cell death may increase the release of tumour-associated antigens with an increase in immune response, inducing tumour cell death [15]. Casares et al. observed this immunogenic effect of anthracycline-treated tumour cells in the absence of any adjuvant or co-stimulus [16]. In addition, one may hypothesize, that this immune effect may be more robust in the presence of tumour cells as is the case in the neoadjuvant setting, as opposed to the adjuvant setting. This might be an argument for choosing neoadjuvant chemotherapy, not using a too high dose of corticosteroids for at least the first doses of chemotherapy. More studies are needed to evaluate the interaction between immuno-surveillance and different types and timings of chemotherapy regimens.

Xing et al. suggested that dexamethasone could suppress immune response by enhancing programmed cell death protein 1 (PD-1) [17]. PD-1 and programmed death ligand-1 (PD-L1) form the PD-1/PD-L1 complex, which plays a role in down-regulating T-cell activity, which may result in faster tumour growth and poor prognosis in the clinical setting of anti-cancer therapy. Hence, it can be hypothesized that dexamethasone may have a negative influence on anti-cancer therapy efficacy through a negative impact on a tumour-related immune response. In our study different dosages of dexamethasone were used to prevent chemotherapy-related allergic reactions and other adverse effects in the two treatment arms. For docetaxel-based treatment 8 mg of dexamethasone orally was given twice daily the day before, of and after docetaxel, whereas during AC treatment 8 mg of dexamethasone intravenously was given shortly before each cycle. Possibly, the upfront use of high-dose corticosteroids during all cycles of concurrently treated arm versus in the last four cycles only of the sequentially treated arm might be a possible explanation for the difference in efficacy between the sequential versus concurrent use of taxanes [1, 9].

As we discussed earlier, the toxicity of both regimes was manageable [1]. The most important difference was the incidence of febrile neutropenia, which was reported in 23% of patients treated with the sequential regimen where primary prophylaxis with granulocyte colony-stimulating factor (G-CSF) was not part of the treatment protocol. This was sharply higher than the 9% rate of febrile neutropenia during TAC chemotherapy where primary G-CSF prophylaxis was mandatory. Most of these febrile events in the AC-T arm occurred during docetaxel mono-chemotherapy (82%). Other studies reported a risk of febrile neutropenia between 5 and 25% [18, 19]. The European Organisation for Research and Treatment of Cancer guidelines considered AC-T chemotherapy to have a high risk of febrile neutropenia (>20%) [20]. For this reason, we would now routinely recommend the use of G-CSF prophylaxis for AC-T chemotherapy during the four cycles of docetaxel chemotherapy [20]. Hence, during AC-T chemotherapy, G-CSF prophylaxis is only required in four cycles instead of six during TAC chemotherapy, which can be considered an advantage for sequentially delivered chemotherapy. More importantly, with AC-T a lower cumulative anthracycline dose can be delivered, which is very attractive as a possible deterioration of cardiac performance has already been reported in patients who received more than approximately 250–300 mg/m^2^ of anthracycline [21, 22]. Limiting G-CSF prophylaxis for four cycles during the docetaxel monotherapy saves costs without an increase of febrile neutropenia, together with the lower cumulative dose per agent compared to TAC chemotherapy makes the AC-T chemotherapy the most cost-effectiveness approach in times of rapidly rising healthcare costs. Finally, with AC-T chemotherapy a cold cap to prevent hair loss may be used. The Dutch Scalp Cooling Registry reported that of scalp-cooled patients; 63% of patients treated with AC-T chemotherapy did not wear a head cover during their last chemotherapy session in contrast to 8% of patients treated with TAC chemotherapy [23].

To conclude, we showed that sequential AC-T neoadjuvant chemotherapy outperformed concurrent TAC chemotherapy in non-metastatic breast cancer patients, given at a lower cumulative dose.

## Appendix

See Table 1.
